# Have we forgotten the non-COVID-19 diabetic patients? Impact of lockdown on daily life, sleep and mental health: cross-sectional study in Moroccan diabetic patients

**DOI:** 10.11604/pamj.2022.42.134.30475

**Published:** 2022-06-17

**Authors:** Soukaina Laidi, Nassim Essabah Haraj, Siham El Aziz, Asma Chadli

**Affiliations:** 1 Mohammed VI University of Health Sciences, Casablanca, Morocco,; 2 Endocrinology, Diabetology and Metabolic Diseases Department, CHU Ibn Rochd, Casablanca, Morocco,; 3 Laboratory of Neurosciences and Mental Health, Casablanca, Morocco,; 4 Faculty of Medicine and Pharmacy-University Hassan II, Casablanca, Morocco

**Keywords:** Lockdown, diabetes, sleep, mental health

## Abstract

The main objective of this work is to describe the impact of lockdown on daily life, sleep and the mental health of Moroccan diabetics patients. The secondary objective is to study the factors affecting compliance with lockdown and deterioration of sleep in diabetic patients while lockdown. It´s a cross-sectional study including diabetic patients followed up at the Endocrinology department of Casablanca. Patients answered a questionnaire about Socio-demographic characteristics, Sleep-related characteristics and predominant activities during the lockdown. The psychological impact was assessed by the score of Anxiety and Depression Assessment Scale (HDAS). Statistical analysis was carried out using SPSS 20 software. Bedtime was shifted by 53 minutes during the lockdown. Waking time was also 1 hour 18 minutes later, while sleep duration increased from 8 hours 20 minutes before lockdown to 8 hours 30 minutes during it (p=0.24) with a deterioration in sleep quality reported by 53 patients. Sleep deterioration was not correlated with anxiety or duration of screen exposure, but was more related to age between 50 and 65 years old. HAD score showed anxiety in 29 patients which were correlated to the female gender. The study included 100 patients with an average age of 48 years. 38 patients had a professional activity before lockdown, 5 of them kept working face-to-face, 3 teleworked and 30 were unemployed. Only 59 % of them respected lockdown. This respect was correlated with female gender, educational level and the number of members under the same roof when it is more than 6. Deterioration in sleep, a change in bedtime and waking time and an increase in anxiety was observed in diabetic patients during the lockdown. Therefore, the psychiatric care system needs to adapt to provide psychological support not only to infected persons but also to other vulnerable communities including diabetic patients.

## Introduction

In the context of COVID-19 pandemic, the media and scientific literature are focusing attention on the evolution of the pandemic, the psychological impact of quarantine and strategies to reduce it [1]. Indeed, lockdown is an anxiety-provoking period due to fear of contamination, professional difficulties, loss of reference points and changes in daily activities. All of these factors can have significant psychological repercussions and potential disruption of sleep, since sleep plays a central role in maintaining our mental health and our general state of health [2]. In addition, sleep is involved in the modulation of immune parameters essential to host resistance [3,4], a phenomenon that appears to be crucial in this context of response to a virus. The diabetic patients have in addition to the anxiogenic factors mentioned above another added factor which is the management of his disease in these conditions where access to the health care system is limited for non-COVID-19 patients because of endogenous (the patient´s fear of being contaminated) and other exogenous (the saturation of some hospitals by COVID-19 cases) barriers. The objective of this work is to describe the impact of lockdown on the daily life, sleep, and mental health of Moroccan diabetics patients.

## Methods

**Study type:** this is a cross-sectional study, carried out at the Endocrinology and Metabolic Diseases department of the Ibn Rochd University Hospital of Casablanca during the lockdown in Morocco between the 1^st^ and the 10^th^ June 2020 including patients´ with diabetic type 2 over 18 years old and diabetic type 1 over 14 years, followed or had been hospitalized in the department. Non-consenting patients and patients who did not respond to questionnaires were excluded from the study.

**Data collection method:** clinical-biological information before the lockdown was collected from medical records: age, gender, type of diabetes, duration of diabetes, treatment, glycated haemoglobin. All patients were contacted by phone during lockdown between the 1^st^ and the 10^th^ of June we were not able to convene all patients in person because of the pandemic, so we opted for the remote mode. After informed consent was obtained, patients answered individually a questionnaire about housing, number of members living in the same house, sleep duration, time of bedtime and time of getting up, quality of sleep, duration of exposure to screens, respect of lockdown and predominant activity during it. Patient beliefs were assessed using the following questions: is the disease more severe in people with diabetes? Is the disease fatal in diabetics? Are you more at risk of getting the disease than others? Anxiety and Depression Assessment Scale score (HAD) was calculated to estimate the psychological impact.

**The variables studied were:** socio-demographic characteristics: age, gender, education level, marital status, number of persons living under the same roof, housing, and work arrangements during confinement; diabetes-related characteristics: glycated haemoglobin; lockdown characteristics: respect for the lockdown was defined by a maximum of 2 exits per week, and predominant activities during the lockdown; sleep-related characteristics: sleep duration, bedtime and wake-up time, quality of sleep, psychological impact using the HAD score (Anxiety and Depression Rating Scale).

**Statistical analysis:** data entry was performed at the end of the collection on SPSS 20 software. Data analysis: statistical analysis was performed by SPSS20.0 software in the first step, we proceeded to a descriptive analysis of the socio-demographic and clinical characteristics of the patients which will be represented in the form of a table. The quantitative variables (age, duration of diabetes...) were described in terms of means and standard deviation. The qualitative variables (profession, marital status, level of education...) were described in terms of percentages. In order to study interactions between different variables, we used inferential statistical techniques. For the cross-tabulation of qualitative variables, we used the Chi-square test or Fisher´s exact test if the conditions for the application of Chi-square were not valid. In addition, we used tests of comparison of means when the Inferential Statistics variables were quantitative. In this case, we used the Student test when the variances were homogeneous, otherwise, we used the non-parametric Mann-Whitney test. The IBM SPSS Statistics 20 program (IBM Corp, Armonk, NY, USA) was used for the statistical analyses, and p < 0.05 was considered the limit of statistical significance.

**Ethical approval:** all information collected on individuals was kept confidential and anonymous. Study data were stored in a secure location. The consent of all patients was obtained orally because patients were contacted by phone and they answered a questionnaire by phone.

## Results

**General characteristics of the population:** the study involved 100 patients with a female predominance: 57 women and 43 men. The mean age of our patients was 48±16 years. Before lockdown, 38 patients were active full-time, 5 of them kept a face-to-face job, 3 patients switched to telework and 30 patients became unemployed. Socio-demographic characteristics of the participants are shown in Table 1. The average duration of diabetes was 10.6 years±6.90 (from 6 months to 47 years). Type 2 diabetes was the most present in the study (71%), while 27% of patients had type 1 diabetes and 2% had diabetes secondary to prolonged corticosteroid therapy. The average HbA1c before the lockdown was 8.98±2.28.

**Table 1:** socio-demographic characteristics of the participants during the lockdown

Variable	N (%)
**Age**	
14-30	18 (18)
31-50	30 (30)
50-65	35 (35)
>65	17 (17)
**Gender**	
Female	57 (57)
Male	43 (43)
**Marital status**	
Single	25 (25)
Married	67 (67)
Divorced	4 (4)
Widower	4 (4)
**Educational level**	
Illiterate	37 (37)
Primary	16 (16)
College	20 (20)
High school	20 (20)
University	7 (7)
**Number of people living together**	
1 -2 persons	10 (10)
3 -4 persons	22 (22)
5 -6 persons	36 (36)
7-10 persons	29 (29)
>10 persons	3 (3)
**Housing**	
Apartment	56 (56)
House	38 (38)
Shared room	6 (6)

**Experience of lockdown:** lockdown compliance: lockdown was respected by only 59% of patients (maximum 2 exits per week). Compliance with lockdown was not correlated with the number of family members nor with smoking but it was correlated with gender and educational level. We observed that patients with doubtful anxiety or certain anxiety respect more lockdown but not statistically significant (Table 2). Activities during the lockdown: the predominant activities during this period were watching television in 40% with an average of 4 hours per day, using a smartphone in 20% with an average of 5 hours and 48 minutes per day, and cooking in 20% and reading books in 5%.

**Table 2:** factors associated with the lockdown compliance

Variable	No compliance with lockdown	Lockdown compliance	p
**Gender**			
Male	23	20	0.023
Female	18	39	
**Age**			
14-30	10	8	
31-50	12	18	NS
50-65	12	23	
>65	7	10	
**Marital status**			
Single	12	13	NS
Married	25	40	
Divorced	2	2	
Widower	2	4	
**Educational level**			
Illiterate	9	28	
primary	5	11	0.02
College	10	10	
High school	13	7	
University	4	3	
**Type of diabetes**			
Diabetes type 1	13	13	NS
Diabetes type 2	28	44	
Other types	0	2	
Smoker	6	8	NS
**Housing**			
Apartment	27	29	NS
House	13	25	
Shared room	1	5	
**Number of people living together**			
1 -2 persons	5	5	
3 -4 persons	5	17	
5 -6 persons	21	15	0.058
7-10 persons	9	20	
>10 persons	1	2	
**Anxiety**			
No anxiety	10	6	
Doubtful anxiety	6	15	0.06
Certain anxiety	9	20	

**Sleep characteristics:** bedtime was shifted by 53 minutes during the lockdown. Wake-up time was also 1 hour 18 minutes later, while sleep duration increased from 8 hours 20 minutes before lockdown to 8 hours 30 minutes during lockdown (p=0.24) with a deterioration in sleep quality reported by 53 patients. Deterioration in sleep quality was not correlated with anxiety or duration of screen exposure but was more related to age (Table 3).

**Table 3:** factors related to deterioration of sleep quality in diabetics patients during the lockdown

Variables	Deteriorated sleep	Stable sleep	P
**Gender**			
Male	23	19	NS
Female	30	25	
**Age**			
14-30	10	8	
31-50	13	16	0.02
50-65	25	9	
>65	5	11	
**Marital status**			
Single	14	11	
Married	32	32	
Divorced	3	0	NS
Widower	4	1	
**Educational level**			
Illiterate	20	15	
Primary	10	6	NS
College	11	9	
High School	11	9	
University	1	5	
Smoker	5	9	NS
**Housing**			
Apartment	26	27	
House	23	15	NS
Shared room	4	2	
**Number of people living together**			
1 -2 persons	7	2	
3 -4 persons	15	7	
5 -6 persons	17	17	NS
7-10 persons	13	16	
>10 persons	1	2	
Duration of screen exposure/h	4.22	3.64	NS
**Anxiety**			
No anxiety	9	7	NS
Doubtful anxiety	15	5	
Certain anxiety	16	13	

**Psychological impact of lockdown:** the psychological impact evaluated by the HAD score found certain anxiety in 29 patients and depression in 8 patients. The presence of anxiety was not correlated with the number of family members or glycemic imbalance, but it was correlated to gender (Table 4).

**Table 4:** factors related to anxiety in diabetics patients during the lockdown

Variable	No anxiety	Doubtful anxiety	Certain anxiety	P
Gender				
Male	10	5	15	0.04
Female	6	16	14	
Age				
14 -30	1	5	5	
31 -50	6	7	8	NS
50 -65	8	6	9	
>65	1	3	7	
**Smoker**	4	2	6	NS
**Housing**				
Appartment	9	11	17	NS
House	6	7	11	
Shared room	1	3	1	
**Number of people living together**				
1 -2 persons	1	3	4	
3 -4 persons	3	5	8	
5 -6 persons	9	5	5	NS
7-10 persons	3	6	11	
>10 persons	0	2	1	
**HbA1c before lockdown (%)**	8,50	9,67	9	NS

Beliefs of diabetic patients about COVID-19: when asked if the disease is more serious in diabetics, 96% of patients answered yes, 86% of patients said they think the disease is fatal for diabetics and 92% of them think that diabetics are more at risk of getting the disease.

## Discussion

**Sleep:** our study found that the time of going to bed and waking up changed by 53 minutes and 1 hour and 18 minutes without significant change in sleep duration when more than half of the patients reported sleep deterioration. Quality sleep requires regular schedules: numerous studies on the deleterious effects of social jet lag show the negative effects of these disturbed rhythms on both metabolic and psychiatric levels [5,6]. Sleep is under the control of strong regulation of the circadian clock which is blunted by irregularity of the schedules, which can lead to a progressive shift of the clock, most often towards the evening, because its innate period is greater than 24 hours [7] This shift can be problematic after lockdown resetting the clock can be difficult, with severe drowsiness in the morning and insomnia in the evening. The biological clock becomes less and less flexible with age [8]. It depends on a strong light signal in the morning to update the central clock. Exposure to intense light in the evening has two effects on sleep: firstly, it has a direct stimulating effect on wakefulness systems, which allows users to remain more alert and go to bed later [9]. Second, exposure to light in the evening shifts the biological clock by blocking melatonin secretion, making it possible to wake up later in the morning [10,11]. Our study showed no correlation between sleep deterioration and exposure to light screens. However, we did not evaluate the correlation between sleep duration and exposure to light from the screens.

Deterioration in the quality of sleep in lockdown is an important finding since sleep plays an important role in immune defences. A recent review has shown that the risk and severity of COVID-19 infection are influenced by sleep quality [12]. To compensate for these disturbances, and according to Geoffroy *et al*. [13] it is important to maintain a regular rhythm and to reinforce the synchronization of these rhythms through: light: increasing natural light during the day and especially in the morning Social interactions and sleep schedules: adopt regular and usual bedtime and wake-up times and reinforce authorized social interactions during the day (social networks, telephone, messages). Physical activity: practice regular physical exercise, especially in the morning and avoid physical activity too close to bedtime, which could increase physiological activation and disrupt subsequent sleep.

**Psychological impact:** our study found that 29% of patients suffered from anxiety, which was not correlated with the number of persons under the same roof nor with glycemic imbalance. The consequences of confinement on mental health and well-being are multiple. Lockdown can produce hysteria, anxiety and distress due to factors such as loss of control. This situation may be intensified if families need separation, due to the uncertainty of disease progression, financial losses, and increased perception of risk, which is usually amplified by vague information and inappropriate media communications in the initial phase of a pandemic [1,14]. Another very important aspect is stigma and social rejection in the form of discrimination, suspicion and avoidance by neighbours and insecurity.

## Conclusion

Deterioration in sleep, changes in bedtime and wake-up times and increased anxiety have been observed in diabetic patients during the lockdown. This is why the psychiatric care system must adapt to promote psychological support not only for infected persons, but also for other vulnerable communities, including diabetic patients, and the implementation of community strategies, particularly through listening platforms initially intended for the most vulnerable populations which diabetic patients are a part.

### What is known about this topic

- *A review of the psychological impact of quarantine reported negative psychological effects including post-traumatic stress symptoms, confusion, and anger [1];*
- *The risk and severity of COVID-19 infection are influenced by sleep quality disease progression and glycemic control in diabetic patients have been affected by lifestyle changes caused by lockdown [12];*
- *Patients with SARS reported fear, loneliness, boredom and anger, and they worried about the effects of quarantine and contagion on family members and friends; they experienced anxiety about fever and the effects of insomnia [14]*.

### What this study adds

- *Our study showed a deterioration in sleep quality while lockdown was reported by 53% of patients, wish was not correlated with anxiety or duration of screen exposure, but was more related to age;*
- *Only 59 % of patients respected lockdown; this respect was correlated with sex, educational level and the number of members under the same roof;*
- *Our study found anxiety in 29% of patients; the presence of anxiety was likely correlated to the female gender*.
